# Perceived educational impact of the medical student long case: a qualitative study

**DOI:** 10.1186/s12909-020-02182-6

**Published:** 2020-08-07

**Authors:** Corinne Tey, Neville Chiavaroli, Anna Ryan

**Affiliations:** Department of Medical Education, The University of Melbourne, Level 7 North, Medical Building, Grattan Street, Parkville, Victoria 3010 Australia

**Keywords:** Long case, Clinical assessment, Consequential validity, Educational impact, Qualitative research, Medical students

## Abstract

**Background:**

The long case is a traditional method of clinical assessment which has fallen out of favour in certain contexts, primarily due to psychometric concerns. This study explored the long case’s educational impact, an aspect which has been neglected in previous research.

**Methods:**

Three focus groups of medical students (20 in total) and semi-structured interviews of six examiners were conducted. Cook and Lineberry’s framework for exploring educational impact was used as a sensitising tool during thematic analysis of the data.

**Results:**

Participants described the long case and its scoring as having influence on student learning. Engaging in the activity of a long case had an essential role in fostering students’ clinical skills and served as a powerful driving force for them to spend time with patients. The long case was seen as authentic, and the only assessment to promote a holistic approach to patients. Students had concerns about inter-case variability, but there was general consensus that the long case was valuable, with allocation of marks being an important motivator for students.

**Conclusions:**

This study offers a unique focus on the traditional long case’s educational consequences; the extent of its positive impact would support its place within a program of assessment.

## Background

Medical teachers have long grappled with how best to assess the clinical competence of their students [1]. The traditional long case, used to evaluate medical students for over 150 years [2], is a form of clinical assessment which requires a student to spend approximately 1 h with a patient, unobserved, then to present a summary of their history and examination findings to examiners who ask them questions about the case. Due to its reliance on real patients, the long case is generally used to assess the clinical skills of students in the latter years of their training, once they have entered the clinical environment. With the main strength of the long case being its professional authenticity and its weakness being unreliability [3], numerous modifications to the traditional long case have been proposed (such as direct observation, extended testing time, multiple examiners, a structured marking grid or a combination of these), but further evidence is needed regarding the effectiveness of these changes [4]. Despite considerable variation in the way that long cases are conducted and scored at different institutions [5], there is usually some subjectivity to the marking, with examiner questions introducing an unstructured element to the assessment [4]. Though viewed ambivalently by some medical educators [6, 7], the traditional long case is still used to evaluate the clinical skills of doctors, particularly in Australasia where it is a cornerstone of the Royal Australasian College of Physicians’ clinical examinations [8, 9]. However, placing such an emphasis on the long case is atypical in the current climate. Concerns about its suboptimal reliability and case specificity [7] as well as modern-day pressures like rising litigation and student appeals [10] have contributed to universities emphasising tests which yield reliable, more easily defensible results. This has led to traditional long case assessments being phased out from medical schools around the world [1, 11] in favour of objective structured clinical examinations (OSCEs), with the recent emergence of workplace-based assessment such as the mini clinical evaluation exercise (mini-CEX) as an alternative for the evaluation of clinical competence [12–14].

Although reliability and case specificity are important considerations in clinical assessment, there is increasing recognition of the importance of validity in assessment [15] and broader conceptualisations of validity which incorporate inferences made around scoring, generalisation, extrapolation and implications of assessments [16–18] – with implications arguably the most important yet least studied of the inferences [19]. To redress this imbalance in the reporting of consequences validity evidence, Cook and Lineberry have proposed a helpful framework for organising data on the impact of educational assessments [20]. This scaffold considers the influence of the assessment on various stakeholders, and the effect of classifications arising from use of the score, taking into consideration the intended, unintended, beneficial and harmful impacts. Therefore, the educational implications of an assessment comprise a subset of its consequences validity evidence, which is derived from evaluations of its effects on groups as diverse as students, teachers, schools, patients and the wider healthcare system [20].

The long case has been heavily criticised in the medical education literature [5, 7, 21, 22], but it is noteworthy that this criticism has been limited in scope, focussing on its perceived psychometric deficiencies [23]. There are sizeable gaps in the body of research into the long case, with very little formal exploration of its educational implications or impact on student learning, other than limited data drawn from questionnaire-based ratings [23–25]. The purpose of this study is to explore the educational impact of the long case as perceived by medical students and examiners, so that medical educators are able to make a fully informed decision about its place in a program of assessment.

## Methods

### Aim

The present study aims to explore the impact of the traditional long case on student learning, from the perspectives of examiners and medical students, in order to determine if this assessment method should have a role in developing and evaluating clinical competence.

### Setting

This research was conducted at the University of Melbourne, which has a four-year graduate entry medical course with an initial pre-clinical year followed by 3 years of clinical training at inner-metropolitan, outer-metropolitan and rural clinical school sites. Throughout their first clinical year (Year 2), students sit three long cases contributing a total of 20% to their summative mark for the year. Long cases are only used in Year 2 and are introduced to students in a graduated fashion, with students having unlimited time to assess a patient and prepare for their first long case assessment (worth 5% of the subject mark). For their second long case, students have a limit of 90 min with the patient and 24 h to prepare for the assessment (5%), while the final long case assessment (10%) is conducted under strict examination conditions, including 60 min to assess a patient whom they have not previously met. All clinical schools organise additional teaching sessions for students to receive feedback on formative long case presentations (the number of sessions depending on the clinical school). Long cases also form part of the clinical assessment hurdle in Year 2, with students required to pass either the multi-station OSCE or the final end-of-year long case assessment in order to progress to Year 3. Students who fail either of the first two summative long cases are flagged and offered remediation at clinical school level; however, it is only the third summative long case which contributes to the clinical assessment hurdle. The summative long cases are scored out of 10 according to a detailed rubric which considers history and examination findings, synthesis/priorities, impact of illness, basic investigational plan and management issues (Additional file 1). The final long case is assessed by two examiners who review each long case patient prior to student assessment.

### Design and participants

Both medical students and clinicians with experience as long case examiners were invited to participate in this interview-based study. Ethics approval was obtained from the University of Melbourne Department of Medical Education Human Ethics Advisory Group (Ethics ID number: 1749366.1).

Final-year medical students based at inner metropolitan clinical sites were invited to reflect on their Year 2 long case experience through focus groups. Participants were approached via email invitation, followed by a brief recruitment presentation delivered on site. A total of 20 medical students (nine males) participated in three focus groups of 6–8 participants. The focus group method was chosen in order to obtain a wide range of views about the educational impact of the long case and to stimulate discussion among students who might otherwise find an individual interview daunting. Students in their final year were selected as the study population on the basis that their internships for the following year had been confirmed by the time of the focus groups, potentially reducing students’ concerns about possible negative consequences related to their participation in the study. Furthermore, with final semester being an ungraded pass/fail semester, all students were reassured that their participation was voluntary and would have no bearing on their ability to graduate or on their future careers. The focus groups were conducted at three different inner metropolitan sites due to reasons of proximity to the research team; however, there were student representatives from all clinical school zones (inner-metropolitan, outer-metropolitan and rural) in this study.

Six examiners (three females) were recruited through purposive and snowball sampling of senior medical staff. We contacted the directors of medical education at each clinical school site and sought examiners with a minimum of 3 years’ experience examining medical student long cases, a minimum of 3 years’ experience in regular small-group teaching of medical students, and who actively worked with junior medical staff in the hospital setting (to ensure that they would be familiar with the work of a junior doctor). At the conclusion of each semi-structured, face-to-face interview, examiners were asked to nominate others likely to meet our recruitment criteria. Noting that clinicians who disliked long cases might not continue to examine them, we accepted examiners who had historical experience assessing medical student long cases; even so, it became evident that examiners had largely positive opinions of the long case. To achieve greater balance, a concerted effort was made to identify examiners with diverse views, but no-one was identified as holding a predominantly negative view of the long case (despite purposeful attempts to recruit such individuals to the study). Four examiners were physicians and two were surgeons; their time in specialist practice ranged from 11 years to over 30 years.

### Data collection

Students and examiners were each asked five questions (Additional file 2) designed to elicit their views on the long case and its educational impact. Questions were developed by the research team to explore the ways in which the long case may have influenced student approaches to learning during medical school, assess for more far-reaching effects leading into internship, and obtain general views on the long case as an assessment method. The same question sets were used throughout the study, with no additional questions being identified through the data collection process. All interviews were conducted by CT with focus groups aided by an administrative coordinator. Focus groups and interviews were audio-recorded and transcribed verbatim.

### Research team

The research team has experience in medicine and clinical supervision (CT & AR), qualitative research (NC & AR) and health professions education (CT, NC & AR). Whilst AR and NC have limited direct contact with medical students, CT has direct supervisory responsibility for students at one clinical school site; given the potential impact of power differential, the focus group at this site included students from other sites who were visiting on placement. Two of the three focus groups were conducted at sites where CT does not have a teaching role. All members of the research team had pre-existing positive opinions of the long case but were also aware of its limitations. Prior to the focus groups, CT underwent training in qualitative interviewing as part of a Master of Clinical Education. During each interview, CT was careful not to share her own views of the long case with participants and made it clear that negative as well as positive views of the long case were being sought.

### Data analysis

Thematic analysis was selected as the framework for data analysis, owing to its flexibility, accessibility and recognition as a valuable research method in its own right [26, 27]. CT coded all nine transcripts, utilising a complete coding approach to the data. AR and NC reviewed all of the nine transcripts and independently analysed three transcripts each, then met with CT to discuss the coding structure. In case of disagreements, extensive discussion was used until consensus was reached. Once coding was complete, and the process of thematic analysis had commenced, it became apparent that the overarching themes resonated with Cook and Lineberry’s framework for evaluating the impact of educational assessment [20] – considering the impact of the activity and the impact of the scores. Intended, unintended, beneficial and harmful implications of the long case were also considered during data analysis. In the present study, the educational impact of the long case was defined by these implications, that is, the various ways in which summative long cases affected medical students and their learning. Data saturation was achieved after six examiner interviews, with no themes revealed in the final two interviews that had not already been identified in the preceding three focus groups and four examiner interviews.

## Results

Study results were organised around two overarching themes, with both themes being viewed through the perspective of the student and the examiner:

(i) Impact of the long case process
(ii) Impact of the long case score

A further six themes were inductively identified from the dataset, with three sub-themes for each overarching theme.

### Theme 1: impact of the long case process

Exploration of the educational impact of the long case process generated three common themes: the development and refinement of core skills in clinical medicine (‘Fundamentals’); the authenticity of the long case (‘The genuine article’); and the travails of students as they strove to achieve mastery of the task (‘Reaching the pinnacle’).

#### Fundamentals

Students frequently commented that the long case provided them with an organisational structure for approaching and interacting with patients. They spoke of the ways in which they relied on this structure, such as on return to the ward after a research semester; the long case framework had been so ingrained that students could still call it to mind:

[Student 15] “Even just going into the ward, though – I remember when I first went back, how long ago, and I was like, oh! What do I need to do? Just totally blanked. And then the structure of the long case came back … and that helped.”

Students also noticed significant improvements in their ability to manage time when talking to patients, as demonstrated by Student 4’s observation that “… [the long case] was helpful to try and refine that kind of time management skill.”

Though examiners certainly recognised that the long case process was helpful in providing students with a means of “approaching medical clerking in a practical way” [Examiner 1], they also reflected that the long case was the only clinical assessment to test a holistic approach to patients. This made full advantage of the case specificity inherent within the long case, as each patient needed to be treated as a distinct individual.

[Examiner 3] “ … it is a KPI [key performance indicator] of a doctor, really, you know, your ability to do a thorough assessment of the whole of a person and sort out all of their problems and how each problem interacts with the other, and the psychosocial impact of the person’s circumstances and how that impacts on their illness and their ability to comply with the treatment strategy and all that kind of stuff, is only to be got through a long case.”

Where students typically provided concrete examples of skills aided by the long case, examiners tended to make expansive statements about the benefits of the long case, with descriptions such as “it is fundamental in what we all do” [Examiner 4], “exactly the skills that you need as an intern” [Examiner 1] and “teaching them the skills that they need to practise” [Examiner 5]. This implied that the completion of a long case may have demanded so many foundational competencies that it was easier or more natural for examiners to describe the benefits of the long case in broad terms rather than targeting specific domains.

#### The genuine article

Students viewed the long case as an authentic task and contributed many specific examples of the ways in which long cases would assist them during internship, including at patient handover, during preadmission clinic, and when admitting new patients from the Emergency Department, which was described as “just doing a mini long case” [Student 18].

Although a minority of students felt that the long case was somewhat artificial, in that “doctors never go and see patients without knowing anything about them” [Student 9] and that “in real life you probably wouldn’t be that comprehensive” [Student 16], students generally agreed that “it felt legit [sic]” [Student 6] with statements such as “it feels real, cause you come up with an issues list, and management” [Student 10] and “the long case was the only time I felt like I was actually learning to do medicine” [Student 6].

[Student 1] “It’s like all of medicine – there’s two types of study. There’s study for the exams, and there’s study for being a good doctor and a competent doctor. And the two don’t necessarily seem to match up … I do think it falls into that ‘how to be a good doctor’, as opposed to ‘how to do the exams well’.”

In contrast to students’ beliefs that long cases were an authentic representation of medical practice, several students held fairly cynical opinions about the authenticity of the OSCE, which was described pejoratively as a “dance” [Student 8], “very artificial” [Student 12], and “terrible because I feel like you just go in there and you’ve rehearsed a little thing you’re going to do and you just regurgitate it” [Student 11].

One student contrasted the two forms of clinical assessment as follows:

[Student 4] “I really can’t imagine having gone through medical school without having to, I guess, learn skills that I got from trying to practise the long case. I mean, the OSCE’s an entirely academic exercise, and at the bare minimum you don’t really have to think about what you’re doing, you just go through the motions – but the long case you’re forced to process what it is you’re doing, why you’re listening to the heart, or if you’re hearing anything, and then formulate it and discuss it and present it … I think those skills are important. I’m glad that I’ve had the time to work on them in a safe kind of environment.”

Examiners were universal in their opinion that the long case mirrored what doctors do in their everyday work:

[Examiner 1] “I think it directly relates more than most of what you do in the medical course to your job as an intern.”

[Examiner 4] “ … essentially that’s what we do day in day out, is do long cases, or medium-long cases, with almost every patient we meet.”

[Examiner 5] “ … all we’re really testing is what you do every day in your practice.”

[Examiner 6] “it mimics real life”

#### Reaching the pinnacle

This subtheme reflected the conceptualisation of the long case as a summit of sorts, which was challenging to scale and required great perseverance to conquer. Students described the long case as a “stretch goal” [Student 11] and “the pinnacle of what we were trying to do” [Student 8].

[Student 7] “I think there’s no substitute for it. You can’t really assess it any other way as comprehensively as you do in the long case, cause … it’s the culmination of everything, it’s your knowledge and your skills and your ability to relate to a patient and talk to them but also manage time, yeah.”

Students found the long case to be highly complex and challenging, and a minority were deterred from seeing patients if they had insufficient time or energy to undertake such a comprehensive patient assessment:

[Student 9] “ … it was almost like, if I saw a patient and I didn’t do a whole long case, like I’d failed in that interaction? … and so then the follow-on effect of that was like, if I didn’t feel like I had it in me within an afternoon to spend a whole hour, hour and a half with a patient, then I might just stay in the library.”

Examiners also acknowledged the challenging nature of the long case assessment, with Examiner 5 describing it as “the apex of all those other tasks”. Examiners were united in their view that the high educational value of the long case meant it should be retained despite its challenges:

[Examiner 2] “I’ve always told the students: the students that do the best are the students that talk to the most patients, examine the most patients, see the most patients, spend the most time in the wards … patients teach you medicine, not textbooks. And I think the long case in some ways exemplifies that learning and that teaching.”

### Theme 2: impact of the long case score

Challenges associated with standardisation and scoring (‘Comparing apples and oranges’), the emotional impact of their graded long case assessments (‘Emotional roller-coaster’) and the meaning which students and examiners attached to long case scores (‘On/off the mark’) were recurring themes with implications for student learning.

#### Comparing apples and oranges

Students and examiners were aware that the long case could not be fully standardised. Students were preoccupied with the equivalence of assessments at different sites or with different patients, whereas examiners were mostly concerned about what they could do to make it fairer for the students.

[Student 2] “I think because all of that variability exists, it’s a really good assessment in making us do it, but it would seem unfair if it was – it had a large weight.”

[Examiner 4] “ … you obviously bring in the very subjective aspects of patient cases, where some cases will be deemed to be seen as easy and some will be deemed to be more difficult, and is that fair? But I think examiners often take that into account as well, when they’re seeing how they do it.”

[Examiner 5] “ … you’ve got an hour and you get one patient and if that’s not your particular thing or the thing you didn’t study as well as something else, then you could be somewhat, perhaps, at a disadvantage, by almost random circumstance. Of course, that depends on the way that you assess it too. I mean if you – the assessment’s not just about that particular case but on the generalities of the process, you can get around that to a degree.”

#### Emotional roller-coaster

When reflecting on their long case assessments, students typically experienced strong emotions that ran the gamut of high stress, anxiety, apprehension, confidence, enjoyment and accomplishment:

[Student 6] “I found it a really enjoyable assessment in the end. Even though I was obviously stressed by it.”

[Student 11] “ … I found the whole thing really stressful but I also think it’s probably our best assessment, even though I hated it a lot.”

The strongly negative reactions which long cases triggered in some students were mostly related to a fear of the assessment, or of failing it. Students described long cases as “daunting” [Student 13], “intimidating” [Student 11] and “stressful” [Student 12], and spoke of the pressure that they felt from their clinical schools and peers to practise long cases so that they would be prepared for their assessments. Though such profound feelings of fear may be an unintended consequence of assigning scores to clinical assessments, student anxiety about the long case *did* motivate them to engage in a number of positive learning behaviours such as seeing patients on the wards and discussing cases with clinical supervisors.

The students’ emotional upheaval in undertaking summative long cases went largely unrecognised by examiners, who on the whole did not mention the affective sequelae for students.

#### On/off the mark

The meaning ascribed to the long case score by students and examiners was an important concept which became apparent across the focus groups and interviews. There was general agreement that long cases had to be graded for students to “take it seriously” [Examiner 6]. Some students volunteered that if long cases were formative rather than summative, they would have “put much less effort in” [Student 3] and there would have been “decreased motivation” [Student 18]. Although a small number of students wished for long cases to be purely formative as it was “such a rich learning experience” [Student 7] in itself, they had great insight into the fact that their more results-focussed peers would be far less motivated to tackle the long case if it did not count towards their final marks:

[Student 10] “ … some people would fully rise to the occasion, because at the end of the day you just want to be a good doctor and you want to impress the doctors that you work for. But other people would fall so far short of jumping that hurdle and would just be like, oh, I don’t care about that, I’ve got to get whatever they mark OSCEs out of, 40 out of 40 for my hypertension OSCE dance [laughter from group].”

As a point of differentiation from the earlier subtheme ‘The genuine article’, which was centred around the long case’s authenticity, the present subtheme focusses on perceptions of the long case’s validity in assessing clinical competence. Though students and examiners were wary of placing too much weight on a single long case assessment (see ‘Comparing apples and oranges’), both groups felt that student performance in long cases was a true reflection of their present and future clinical competence:

[Student 9] “ … I did quite well in OSCEs and I do, I always do well in written exams, and out of all those things, my long case was the worst … I think that was more reflective of the level I was at clinically, than the exam marks which were great but probably really unreflective of how I was going as a whole doctor.”

[Student 11] “ … it’s just so in depth that you can’t really, you can’t accidentally do really badly or really well”

[Examiner 3] “I think it is the best yardstick against which to measure a student’s performance as a practising doctor”

Examiners in particular regarded competence in the long case as a marker of future clinical competence, asserting that a student proficient in long cases would become a capable intern:

[Examiner 2] “Well I think it certainly reflects their practice as interns. I think there’s no doubt about that – if someone can do a long case, whatever that means, as a student, they’re going to be a good intern, most likely, because they’re going to know the questions to ask, they’re going to know how to follow the leads, they’re going to empathise with the patient, and once you develop empathy and rapport with the patient, you’re much more likely to get a deeper understanding of what this illness or this group of illnesses means to that patient. And I think that once you understand all of that, your treatment plan is going to be persuaded by what you learn about the patient, not just what you know about the disease, but what you learn about that person.”

Observations such as these afforded the long case a certain gravitas and elevated it from the daily grind of student assessment, as it shaped into a means to predict the intangible: whether someone would become a good doctor.

## Discussion

This study aimed to shed light on the educational impact of the long case, as perceived by students and examiners, and to clarify its place in a program of assessment. Viewed through the lens of Cook and Lineberry’s framework for organising consequences evidence [20], it was clear that long case assessments had the important effect of motivating our student informants to spend time with patients. With respect to negative implications of the long case for student learning, the present study reinforced that standardisation and scoring of long cases is challenging [23], and that summative long cases induce feelings of anxiety in students [28]. Although long cases acted as a powerful motivator for students to see patients on the wards, the unintended “flip side” was that a small number of students appeared to deprioritise opportunities for brief patient interactions which fell short of a full-length long case. This, coupled with the tendency for students to find long cases very daunting when first starting out, is indeed a potential negative aspect of the long case, and confirms the importance of scaffolding for complex clinical tasks, such as reinforcing the concept that time spent with patients (regardless of duration) is essential for developing their skills. It was widely acknowledged by examiner informants that the variability inherent within the long case limits its use as a high-stakes barrier examination to some degree; even the strongest supporters of the long case within the examiner group felt that a single long case should only have a relatively low weighting in summative assessment. Even so, recent programmatic approaches to assessment have created a new perspective on accepting and strategically using assessments which may be less robust from an exclusively psychometric perspective [29]. Programmatic assessment rightly shifts the focus onto the whole of the assessment system, so that an individual method which may have limited reliability due to patient variability may yet provide important learning experiences and integrative approaches [30]. Though our institution currently has a clinical assessment hurdle in place, we acknowledge that the theoretical underpinnings of programmatic assessment include the need to consider all results when judging a candidate summatively, as opposed to a reliance on barrier examinations.

Despite the well-documented shortcomings of the long case assessment method [7], the results of this study suggest that the traditional long case has an important positive impact on student learning. Examiners advocated strongly for its retention and drew many parallels between the long case and the work of doctors. It was also common for examiners to express the view that long case performance was a good predictor of future clinical competence, or of students going on to become “good doctors” – a concept which, to our knowledge, has not previously been examined in the long case literature. It was interesting that none of the students in this study volunteered the suggestion that the long case should be removed; despite some negative experiences and misgivings about its inherent variability, there was general agreement that it provided an authentic and valuable learning experience – particularly when compared to the mainstay of student clinical assessment, the OSCE. Examiners largely focussed on the authenticity of the long case without mentioning the ways in which it might differ from usual clinical practice. A potential explanation could be that examiners had a broader perspective on the clinical assessment of medical students (which invariably carries a veneer of artificiality, as genuine workplace-based assessment is difficult to achieve in a student cohort with restrictions on its scope of practice), coupled with greater experience of situations when they might have had to perform a comprehensive patient review without the benefit of investigations or collateral information. The long case fostered a multitude of essential skills in medical students, and the manner in which it tested a holistic approach to patient care was unparalleled in their program of assessment.

Limitations of the study included the possibility that an unusually reflective group of student participants with largely positive views of the long case had been self-selected, and that it would have been helpful to have interviewed at least one examiner with a predominantly negative view of the long case (though the inability to identify a clinician with negative views was informative in itself). The patient voice was also missing from this study; the impact of this time-consuming and at times intrusive assessment on the patients themselves merits further evaluation. It is important to recognise that the conclusions derived from this study are reflective of the manner in which our institution conducted clinical examinations like long cases and OSCEs, hence caution is required when applying these findings to other contexts. Finally, with examiners recognising the resource- and labour-intensive nature of organising long case assessments, a follow-up study examining the cost-benefit ratio would be beneficial from a practical and economic standpoint.

This study is significant because, to our knowledge, it is the first to examine the educational impact of the traditional long case in detail, investigating the perspectives of students and examiners. Their affirmation of the long case as an educationally valuable tool laden with potential for student learning is in stark contrast to a litany of negativity about the long case in the literature, with numerous publications referring to its impending or actual death [6, 7, 21, 31]. Though the long case may be “ancient” in the field of medical education, its holistic nature and benefits for student learning should give pause to those calling for its extinction. Instead, we should be focussing on its validity, especially consequential, and looking at bringing it back from the brink – albeit with a relatively small summative component and a heavy emphasis on feedback and teaching.

The results of this study also have implications for the way in which long cases are introduced to medical students, who found these assessments to be stressful and daunting – even overwhelming at the beginning. Though beyond the scope of the present study, the magnitude of the clinical task would suggest that long cases should be introduced in a graduated and highly supported manner, with students being afforded the opportunity to develop core skills over time and observe exemplar performances such as those of junior doctors preparing for their specialist examinations.

## Conclusions

This research into student and examiner perceptions of the long case provides empirical evidence that the long case can exert an important and beneficial effect on student learning, including acting as a strong motivator for students to engage with patients on the wards, fostering many fundamental clinical skills, and being regarded as having a high degree of authenticity. The long case is not without its weaknesses: it is a complex task which is challenging to score and standardise, and even though it serves as a powerful driving force for students to spend time with patients, its daunting nature may paradoxically deter some students from going to the wards if they feel they have insufficient time or energy to undertake a detailed assessment. There is also the issue of case specificity, where serendipity may contribute to a student’s successful performance. However, the present study suggests that the long case is unique among assessments in promoting a holistic approach to patients – an important consideration given the focus on patient-centredness in contemporary medical education.

In conclusion, the long case appears to provide critical formative skill-development in the clinical encounter, and its positive educational impact, as perceived by students and examiners, would suggest that it does have a place in a program of assessment. Further research is needed to determine how an instrument with such rich educational potential can be optimised for the advancement of student learning.

## Supplementary information

**Additional file 1.** Marking rubric for long cases at the University of Melbourne. Marking rubric used for the summative assessment of Year 2 long cases at the University of Melbourne

**Additional file 2.** Student focus group questions and examiner interview questions. Questions used in medical student focus groups and semi-structured examiner interviews

## Data Availability

The data that support the findings of this study are available from the corresponding author on reasonable request. The data are not publicly available due to their containing information that could compromise research participant privacy.
