# Supplementary material for: Perceived educational impact of the medical student long case: a qualitative study
Source: BMC Med Educ. 2020 Aug 7;20:257. doi: 10.1186/s12909-020-02182-6 (PMC7414530; doi:10.1186/s12909-020-02182-6)
Supplement: Supplementary file 1 — Additional file 1. Marking rubric for long cases at the University of Melbourne. Marking rubric used for the summative assessment of Year 2 long cases at the University of Melbourne [file 12909_2020_2182_MOESM1_ESM.pdf]

ADDITIONAL FILE 1

File name: Additional file 1

File format: .pdf

Title of data: Marking rubric for long cases at the University of Melbourne

Description of data: Marking rubric used for the summative assessment of Year 2 long cases at the University of Melbourne

Marking rubric for long cases at the University of Melbourne

Please rate the student against the standard you would expect of a student at the end of their first year of clinical training.

Please circle **one** performance level only for each domain, then provide **a final judgement** about the student's overall performance **and assign an appropriate score** out of 10 in the box below.

An explanation of the domains is given on the reverse of this assessment form.

**History**	Unsatisfactory	Satisfactory	Good	Excellent
**Examination**	Unsatisfactory	Satisfactory	Good	Excellent
**Synthesis / Priorities**	Unsatisfactory	Satisfactory	Good	Excellent
**Impact of Illness**	Unsatisfactory	Satisfactory	Good	Excellent
**Basic investigational plan and management issues**	Unsatisfactory	Satisfactory	Good	Excellent
**Overall performance**	Unsatisfactory	Satisfactory	Good	Excellent
Score ranges	1 – 4	5 – 6	7 – 8	9 – 10

**Global Score (Out of 10):**	

Criteria for assessment of performance in long case

These are suggested criteria for standards of performance. A student does not need to display all criteria in a section.

Level of Performance	History	Examination	Synthesis, priorities and impact of illness	Basic investigational plan and management issues
**Excellent performance**<br><br>**Score range: 9-10**	• Identifies all major aspects of history<br>• Clearly demonstrates a hypothesis led history<br>• No prompts needed to clarify details<br>• Identifies the relevant negative aspects of history	• Identifies all important physical signs and uses these to support the hypotheses identified in the history<br>• Identifies the relevant negative signs	• Identifies all major and minor problems<br>• Arranges problems in the ideal order of priority<br>• Identifies all of the major psychological and social aspects of the patient's problems	• Suggests all appropriate investigations<br>• Discusses how the results could affect the patient's management
**Good performance**<br><br>**Score range: 7-8**	• Identifies most major aspects of history<br>• Shows elements of a hypothesis-led approach<br>• Minor prompting needed to clarify details<br>• Several relevant negatives considered	• Identifies all important physical signs but does not directly relate these to the hypotheses identified in the history<br>• Identifies some relevant negative signs	• Identifies all major problems<br>• Arranges problems in a suitable order of priority<br>• Identifies most of the psychological and social aspects of the patient's problems	• Suggests several appropriate investigations<br>• Shows awareness of how the results could affect the patient's diagnosis
**Satisfactory performance**<br><br>**Score range: 5-6**	• Identifies several main aspects of history<br>• Minimal evidence of a hypothesis-led approach<br>• Needs substantial prompting to clarify details<br>• Considers few and/ or marginal negative aspects of history	• Identifies most physical signs, but without relating these to hypotheses	• Identifies several problems including most major problems<br>• Arranges problems in a reasonable order of priority but with some problems missing or significantly out of order<br>• Identifies several psychological and social aspects of the patient's problems, including some major problems	• Suggests some appropriate investigations but with some unnecessary but not contraindicated investigations<br>• Can explain how the results might affect the patient's diagnosis, after prompting
**Unsatisfactory performance**<br><br>**Score range: 1-4**	• Identifies few relevant key aspects of history<br>• Omits most important aspects from the history<br>• No evidence of a hypothesis-led approach<br>• Needs significant prompting to consider important details, which may not elicit the required details	• Identifies few relevant signs	• Fails to identify several major problems<br>• Presents an unsuitable order of priority, especially for major problems<br>• Requires substantial prompting to consider relevant problems<br>• Identifies few relevant psychological and social aspects of the patient's problems after prompting	• Suggests investigations which are irrelevant and/ or contraindicated<br>• Minimal awareness of how results could affect patient diagnosis
