# Supplementary material for: Perceived educational impact of the medical student long case: a qualitative study
Source: BMC Med Educ. 2020 Aug 7;20:257. doi: 10.1186/s12909-020-02182-6 (PMC7414530; doi:10.1186/s12909-020-02182-6)
Supplement: Supplementary file 2 — Additional file 2. Student focus group questions and examiner interview questions. Questions used in medical student focus groups and semi-structured examiner interviews [file 12909_2020_2182_MOESM2_ESM.pdf]

ADDITIONAL FILE 2

File name: Additional file 2

File format: .pdf

Title of data: Student focus group questions and examiner interview questions

Description of data: Questions used in medical student focus groups and semi-structured examiner interviews

Student focus group questions and examiner interview questions

Student focus group questions:

1. What, if any, impact did long cases have on the way you approached learning on the wards in Year 2?

2. What, if any, impact did long cases have on the way you approached learning through personal study in Year 2?

3. What, if any, impact do you think that doing long cases back in Year 2 might have for your learning in this final semester?

4. What, if any, impact do you think that your long case assessments will have on your clinical practice when you start internship next year?

5. What are your thoughts on whether we should use the long case as a method to assess medical students?

Examiner interview questions:

1. Please tell me what you think about the medical student long case?

2. Have your views of the medical student long case changed over time?

3. What, if any, impact do you think that long cases might have on the way in which second-year medical students approach learning? This includes, but is not limited to, ward learning and personal study.

4. Do you think that the long case might have a longer-term impact for medical students? Perhaps for their learning in their final years of medical school, or their clinical practice as interns?

5. Do you think we should use the long case as a method of assessing medical students? Why or why not?
